# Sex Differences in Quality of Life in Patients with Ischemia with No Obstructive Coronary Artery Disease (INOCA): A Patient Self-Report Retrospective Survey from INOCA International

**DOI:** 10.3390/jcm12175646

**Published:** 2023-08-30

**Authors:** Sachini Ranasinghe, C. Noel Bairey Merz, Najah Khan, Janet Wei, Maria George, Colin Berry, Alaide Chieffo, Paolo G. Camici, Filippo Crea, Juan Carlos Kaski, Mario Marzilli, Martha Gulati

**Affiliations:** 1 Barbra Streisand Women’s Heart Center, Smidt Heart Institute, Cedars-Sinai Medical Center, Los Angeles, CA 90048, USA; 2 Houston Methodist Hospital, Houston, TX 77030, USA; 3 INOCA International, Glasgow G12 8QQ, UK; 4 British Heart Foundation Glasgow Cardiovascular Research Centre, University of Glasgow, Glasgow G12 8QQ, UK; 5 IRCCS San Raffaele Scientific Institute, 20132 Milan, Italy; 6 Department of Medicine, Vita Salute San Raffaele University, 20132 Milan, Italy; 7 Department of Cardiology, Catholic University of the Sacred Heart, 00168 Rome, Italy; 8 Molecular and Clinical Sciences Research Institute, St. George’s University of London, London SW17 0RE, UK; 9 Department of Surgical, Medical and Molecular Pathology and Critical Care Medicine-Cardiology Division, University of Pisa, 56126 Pisa, Italy

**Keywords:** ischemia with no obstructive coronary artery disease (INOCA), ischemic heart disease, sex differences, quality of life, functional capacity

## Abstract

Women with obstructive coronary artery disease (CAD) have a relatively lower quality of life (QoL) compared to men, but our understanding of sex differences in QoL in ischemia with no obstructive coronary artery disease (INOCA) is limited. We conducted a survey of patient members of INOCA International with an assessment of self-reported health measures. Functional capacity was retrospectively estimated using the Duke Activity Status Index (DASI), assessing levels of activities performed before and after INOCA symptom onset. Of the 1579 patient members, the overall survey completion rate was 21%. Women represented 91% of the respondents. Estimated functional capacity, expressed as metabolic equivalents (METs), was higher before compared to after INOCA diagnosis comparably for both women and men. For every one MET decline in functional capacity, there was a significantly greater decline in QoL for men compared with women in physical health (4.0 ± 1.1 vs. 2.9 ± 0.3 days/month, *p* < 0.001), mental health (2.4 ± 1.2 vs. 1.8 ± 0.3 days/month, *p* = 0.001), and social health/recreational activities (4.1 ± 1.0 vs. 2.9 ± 0.3 days/month, *p* = 0.0001), respectively. In an international survey of patients living with INOCA, despite similar diagnoses, clinical comorbidities, and symptoms, INOCA-related functional capacity declines are associated with a greater adverse impact on QoL in men compared to women.

## 1. Introduction

The diagnosis of ischemic heart disease was previously believed to require the presence of obstructive coronary artery disease (CAD). However, there is increased recognition of ischemia with no obstructive coronary artery diseases (INOCA) and myocardial infarction with no obstructive coronary artery disease (MINOCA) among those presenting with symptoms and signs suggestive of ischemia, with a female preponderance [1,2,3,4,5]. Similarly, among patients undergoing elective coronary angiography for stable angina pectoris, approximately 40% are noted to have non-obstructive coronary arteries [6], with women being at least twice as likely to have INOCA compared with men [4]. INOCA has high morbidity, increased risk of major adverse cardiac events, and mortality similar to obstructive CAD [4], especially for those who remain symptomatic [7]. Mechanisms behind INOCA are heterogeneous, including endothelial and non-endothelial dependent microvascular dysfunction and epicardial and microvascular coronary vasospasm [5].

Historically, INOCA has posed a formidable challenge in terms of diagnosis and treatment. Due to the probable involvement of multiple mechanisms behind INOCA, specific phenotyping beyond symptoms and ischemia is essential for improving the understanding of diagnosis and treatment [5]. The most recent ACC/AHA/Multisociety Chest Pain guidelines have recognized INOCA, broadening the definition of CAD to encompass both obstructive and non-obstructive CAD and providing a clinical decision pathway for INOCA [8]. The most comprehensive diagnostic evaluation of INOCA involves invasive coronary vasoreactivity studies [9]. The use of coronary vasoreactivity testing is associated with continual improvement of symptoms and higher quality of life (QoL) [9]; however, they are unfortunately not routinely performed due to various reasons, including lack of standardized protocols [10], lack of awareness regarding evaluation of INOCA, and limited availability of medications for testing. Optimal medical treatment is based on the underlying cause of myocardial ischemia [9]. Consequently, patients with INOCA are often inadequately diagnosed and treated for many years.

Quality of life encompasses various aspects, including physical, emotional, psychological, and social well-being, all of which can be influenced by an individual’s health condition and impact an individual’s ability to live a fulfilling life. Patients with obstructive CAD often report poorer quality of life (QoL), with women experiencing relatively poorer QoL when compared with men [11]. Symptoms of INOCA are characterized by their unpredictability, as they can occur during exertion, rest, or even in the evening or at night. These episodes of symptoms often leave individuals feeling fatigued for days after engaging in activities [12]. As a result, individuals with INOCA may experience limitations in their daily activities, diminished social interactions, and a decline in their emotional well-being. Our understanding of sex differences in QoL in INOCA is limited. We previously reported associations between INOCA and patient-reported QoL [13], and we now seek to determine the sex differences in QoL related to functional capacity changes in terms of self-reported physical, social, and mental health.

## 2. Materials and Methods

The survey was provided to all patient members of United Kingdom (UK)-based INOCA International, a patient education, support, and advocacy group, as previously reported [13]. The survey questionnaire was previously published in our original report [13]. The survey aimed to gather insights into how INOCA had influenced the medical care, health, and overall life of the respondents, and it consists of 49 multiple-choice questions and 1 free-text option for additional comments. A copy of the survey is included in the Supplementary Materials. The survey was released through multiple modalities, such as a newsletter and via social media through Twitter and Facebook, with access to the link limited to patient members of INOCA International. The online survey collection was made available for 2 months, from 27 October 2021 to 27 December 2021, and limited to one entry from a single IP address. All data were collected through SurveyMonkey and anonymized. The approval for the survey was obtained from the Cedar-Sinai Institutional Review Board (Approval Code: STUDY00001724).

Functional capacity was retrospectively estimated in the survey assessing levels of activities performed before and after symptom onset using the Duke Activity Status Index (DASI), a tool previously validated in women enrolled in the WISE Study [14]. The DASI Score was converted to metabolic equivalents (METs) using the following formula: METs = (0.43 × DASI + 9.6)/3.5, as previously described [15]. QoL was assessed by self-report without utilizing a previously validated QoL assessment tool.

The statistical analysis included descriptive and frequency distributions, with chi-squared statistics for categorical variable comparisons and t-tests for continuous variable comparisons. Using XL-STAT Software, survey results for “Did your symptoms change at menopause?” with a choice of “Male (Not Applicable)” were evaluated to identify the sex of the respondent. Independent T-tests were performed on pre- and post-diagnosis METs between men and women. Linear regression analysis was performed on post-diagnosis METs for men and women related to days of declining physical, mental, and combined health per month.

## 3. Results

Baseline characteristics and overall survey findings were previously reported [13]. Briefly, of the 1579 patient members of INOCA International, 328 (21%) respondents completed the survey. Thirty-one respondents reported not having INOCA and were not eligible to answer additional questions, and an additional eighteen respondents who did not complete the “Did your symptoms change at menopause?” question were excluded. Overall, 279 respondents were included in this analysis.

### 3.1. Characteristics of the Respondents

Most respondents were women (91%), with the proportion of women respondents being higher than that of INOCA International (83% women). The baseline characteristics, symptoms, and triggers stratified by sex are included in the Supplementary Tables S1–S3.

The most common reported diagnoses for both men and women were coronary microvascular disease and coronary artery spasm, with no significant differences between the two groups. Migraines and frequent headaches were the most common co-morbid conditions for both men and women. The majority of men (58%) and women (69%) were diagnosed with INOCA between the ages of 40 and 60 years. None of the men reported a previous diagnosis of stress cardiomyopathy or heart failure with preserved ejection fraction.

### 3.2. Medical Evaluation of INOCA

Most men (69%) and women (39%) had experienced symptoms of INOCA for 1–5 years. Furthermore, for the majority (approximately 65% for both sexes), it took up to 3 years from symptom onset to receive a diagnosis of INOCA. Chest pain, pressure, or discomfort was the most common symptom for both sexes. More women experienced gastrointestinal symptoms compared to men, although the difference was not statistically significant. Stress, exercise/exertion, and heightened emotional state were the most common triggers among both men and women. For both men and women, over three-quarters were told their symptoms were not cardiac, and more than half were told their symptoms were due to gastroesophageal reflux disease. There were no sex differences in referral to psychiatry or prescriptions for antidepressants or anxiolytics. Approximately 50% of both men and women were taken to hospital and obtained ECG with cardiac monitoring. There were no sex differences in the total number of consults seen prior to the diagnosis of INOCA or any difference in the number of non-invasive and invasive diagnostic imaging performed prior to making the diagnosis.

### 3.3. Quality of Life Measures

**Figure 1:**
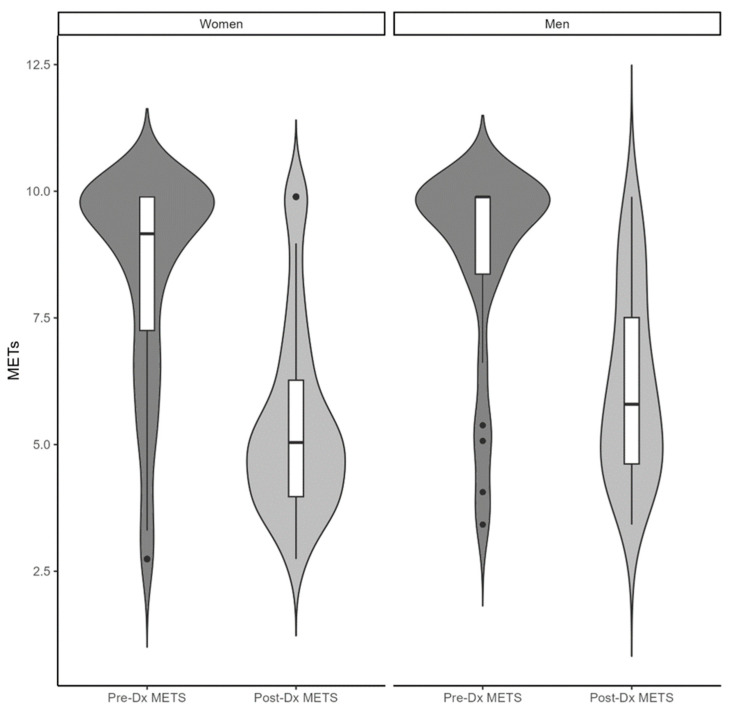
Functional Capacity Before and After Symptom Onset. Functional capacity in respondents with INOCA before and after symptom onset. Legend: Pre-Dx = pre-diagnosis, Post-Dx = post-diagnosis, METs = metabolic equivalents.

While currently living with INOCA, most respondents (58% of men and 51% of women) reported their overall health as being fair or poor. Additionally, functional capacity significantly decreased after the onset of INOCA symptoms, when compared to prior to the onset of INOCA symptoms for both women (8.6 ± 1.8 vs. 5.6 ± 1.8 METs, *p* < 0.00001) and men (8.7 ± 2.0 vs. 6.1 ± 1.8 METs, *p* < 0.00001) (Figure 1). After the onset of INOCA symptoms, only 11% of the women and 19% of the men were able to perform >8 METs. There were no differences in functional capacity between men and women before or after INOCA symptom onset (Table 1).

Both men and women reported that INOCA adversely affected their home life, relationship with their partner/spouse, social life, and sex life. More men reported INOCA adversely affecting their work life and having to reduce work hours due to symptoms. More women reported having to retire early and change jobs to less stressful positions or lower-paying jobs due to INOCA symptoms. A similar proportion of men and women applied for disability. However, there were no statistically significant differences between the sexes in the above findings.

For those living with INOCA, there were sex differences in the association between declines in functional capacity and various aspects of QoL. When comparing men to women, for every one MET decline in functional capacity, there was a greater loss in days of declining health per month for physical health (4.0 ± 1.1 vs. 2.9 ± 0.3 days/month, *p* < 0.001), mental health (2.4 ± 1.2 vs. 1.8 ± 0.3 days/month, *p* = 0.001), and social health/recreational activities (4.1 ± 1.0 vs. 2.9 ± 0.3 days/month, *p* = 0.0001).

## 4. Discussion

Our study illustrates significant sex similarities and differences in patients living with INOCA. Men and women had similar clinical diagnoses, symptoms, triggers, referral patterns, and clinical evaluations. Furthermore, women and men living with INOCA had a comparable decline in functional capacity of approximately three METs after the onset of INOCA symptoms. While this decline in functional capacity was associated with poor QoL in terms of physical, mental, and social health for both sexes, the observed days lost per month in physical, mental, and social health QoL was significantly greater in men compared to women.

After the onset of INOCA symptoms, the percentage of respondents with good functional capacity, defined as an exercise capacity >8 METs [16], was more than four times lower for men and six times lower for women compared to their pre-INOCA symptom levels. In our study, men and women who participated in the survey had similar functional capacity levels prior to the onset of their INOCA symptoms. Previous studies based on cohorts of healthy adults in the general population have shown that men generally have higher functional capacity than women of similar age, with exercise capacity decreasing with age in both [16,17]. While functional capacity has diagnostic and prognostic implications among those with CAD based on cohorts mainly consisting of obstructive CAD [18], there is limited literature on sex differences in functional capacity in this population. The men who participated in the current survey may have been less physically fit prior to the onset of INOCA, or they may have been older than the women prior to the onset of INOCA symptoms. The current age or the age at INOCA diagnosis of the survey participants was not collected in this study. Additionally, it is possible that men with poorer baseline functional capacity may have been more symptomatic and may have been more likely to seek out a patient advocacy group and participate in such a survey. Hence, self-selection could also be a reason for our survey findings. Furthermore, the sex differences in QoL related to functional capacity may be influenced by differences in work environments. For instance, if the men in our study engaged in more physically demanding work activities compared to women, then they may perceive greater limitations in QoL despite similar declines in functional capacity. Finally, physical inactivity may be a risk factor for INOCA, leading to coronary endothelial dysfunction [19].

Previous studies have demonstrated that women evaluated for suspected CAD are more likely to have normal coronaries and non-obstructive CAD but worse QoL [5,20]. Both women and men with non-obstructive CAD can remain symptomatic with relatively high rates of hospitalization for angina and major adverse cardiac events, including heart failure, compared to age-matched asymptomatic individuals [21,22]. The CIAO-ISCHEMIA trial, which looked at a cohort of patients with INOCA to explore changes in angina over one year using Seattle Angina Questionnaire (SAQ) scores, showed that the male sex was associated with a lower likelihood of change in SAQ scores, hence better QoL, which is in contrast with our findings [23]. The population studied in CIAO-ISCHEMIA consisted mostly of women, like the population surveyed in our study. Additionally, a prior international observational cohort of patients with coronary microvascular dysfunction from the Coronary Vasomotor Disorders International Study (COVADIS) group showed that women reported significantly greater physical limitations and lower SAQ scores than men, indicating worse QoL in women [20]. However, this population differed from the currently surveyed population, given that there were 36% men, and all patients in the COVADIS had documented impaired coronary microvascular dysfunction and were being treated at one of 14 specialized centers. Additionally, there was no measure of change in QoL assessment, just an initial assessment at the time of diagnosis. They also did not assess changes in functional capacity and its relationship to changes in QoL. Previous studies that have looked at cohorts with obstructive CAD for both chronic stable angina and after acute myocardial infarction showed poorer quality of life in women based on SAQ scores [11,24]. Our study demonstrated that declines in functional capacity were related to greater declines in QoL in men with INOCA when compared to women. While we did not use a direct measure of QoL, such as SAQ scores, a greater change in QoL in men compared to women with INOCA related to a similar decline in functional capacity has not been previously reported. We speculate whether the observed differences between sexes in our study may be due to gender roles in addition to the biological differences between these groups in genetics and hormones and different vulnerabilities to various disorders, including psychiatric disorders. Psychological stress is associated with adverse cardiovascular outcomes, and coronary microvascular dysfunction and coronary vasospasm are triggered by psychological distress and depression [25]. Women with INOCA have higher rates of anxiety and depression and use benzodiazepines more frequently compared to men [26]. It is possible that treatment of anxiety/depression in women results in an improvement in the overall QoL, which may explain the sex differences in QoL related to a similar decline in functional capacity seen in our study; however, the collection of medical treatment was not part of this survey.

Another reason for the sex differences in QoL could be due to sex differences in INOCA treatment. The CorMicA trial showed that stratified medical therapy based on invasive diagnostic coronary function testing results improves angina in patients with no obstructive CAD [9]. Prior research compared men and women who underwent coronary function testing found a comparable prevalence of coronary vascular dysfunction between the two sexes but highlighted sex differences in phenotype [27]. The men were found to have a higher prevalence of coronary epicardial spasm and a lower prevalence of coronary microvascular spasm, whereas women had similar rates of both types of coronary spasm and also a higher prevalence of impaired coronary flow reserve (CFR) [27]. It is possible that the men who participated in this survey had not had appropriate testing to understand the underlying mechanisms of their INOCA and had received suboptimal treatment for angina. Furthermore, a single-center study showed that women who were diagnosed with coronary microvascular endothelial dysfunction and underwent treatment had a higher QoL based on the 36-Item Short Form Survey (SF-36) compared with women who did not have a diagnosis of microvascular endothelial dysfunction [28]. The same study showed a higher percentage of women with coronary microvascular endothelial dysfunction compared with men, and they hypothesized that the diagnosis and treatment of coronary microvascular disease may have greater benefit on women compared with men [28]. If men and women respond to INOCA treatment differently and also potentially have different diagnoses, this may explain why men in our survey had poorer QoL related to a similar decline in functional capacity after the onset of INOCA symptoms. Again, information about medications was not collected in the survey, but this raises additional questions about whether such differences in treatment response are driven by sex (biological variable) or gender. Therefore, we need prospective studies to further elucidate the sex and gender-specific factors that affect INOCA symptoms, functional capacity, and QoL.

### Study Limitations

The study has several limitations to consider. INOCA is a condition that predominantly affects women. While most of the respondents were women, the proportion of males who participated in the survey was less than the male representation of INOCA International members and was also lower than the established prevalence of INOCA among men [29]. As with all survey-based studies, there is concern that respondents with biases may self-select into or out of the sample. This survey was limited to a support group and thus includes a highly selected group of participants who had an established diagnosis or suspicion of INOCA, had undergone evaluation, and have sought out such an organization, and thus may not be representative of all INOCA patients. Furthermore, the survey was self-reported without documentation or adjudication of the clinical status or diagnoses. The participants’ ages were not collected in the survey, and thus, we were not able to include age as a covariate in the regression analysis. The patients’ assessments of their quality of life and functional capacity before experiencing symptoms of INOCA may be influenced by recall bias. To evaluate the functional capacity of the participants, the DASI questionnaire was employed, which has been validated in populations with ischemic heart disease, including individuals with INOCA, as observed in the WISE study [11]. However, since the survey was conducted online without an interviewer, there was no opportunity to ensure complete comprehension, and the questions were susceptible to various interpretations.

## 5. Conclusions

In an international INOCA survey, despite similar diagnoses, clinical comorbidities, symptoms, and similar pre-diagnosis functional capacity, INOCA-related functional capacity declines are associated with a greater adverse impact on QoL in men compared to women. These findings highlight the need for future studies to incorporate QoL measures and develop tailored INOCA treatment strategies stratified by sex. By doing so, we can aim to optimize patient outcomes and improve the overall well-being of both men and women affected by INOCA.

## Figures and Tables

**Table 1:** Health status and quality of life.

	Men	Women	*p*-Value
Total, N, %	26	253	
Overall Health After Symptom Onset			
Excellent	0 (0)	6 (2.4)	0.4336
Very Good	3 (11.5)	42 (16.6)	0.5636
Good	8 (30.8)	75 (29.6)	0.9303
Fair	11 (42.3)	80 (31.6)	0.4452
Poor	4 (15.4)	50 (19.8)	0.6539
Functional Capacity Level by DASI Score before onset of INOCA symptoms			
<5 METs	2 (7.7)	23 (9.1)	0.8273
5–8 METs	3 (11.5)	58 (22.9)	0.2657
>8 METs	21 (80.8)	172 (68.0)	0.5779
Estimated Exercise Capacity (METs)	8.7 ± 2.0	8.6 ± 1.8	0.7857
Functional Capacity Level by DASI Score after onset of INOCA symptoms			
<5 METs	9 (34.6)	116 (45.8)	0.4845
5–8 METs	12 (46.2)	110 (43.5)	0.8710
>8 METs	5 (19.2)	27 (10.7)	0.2602
Estimated Exercise Capacity (METs)	6.1 ± 1.8	5.6 ± 1.8	0.1785
Mental Health After Onset of Symptoms			
INOCA adversely affected your Mental Health	18 (69.2)	191 (75.5)	0.7876
INOCA Negatively affected your outlook on life	22 (84.6)	185 (73.1)	0.6328
Social Health After Onset of Symptoms			
INOCA Adversely affected home life	21 (80.8)	218 (86.2)	0.8336
INOCA adversely affected your relationship with partner/spouse	14 (53.8)	146 (57.7)	0.8421
INOCA adversely affected your social life	21 (80.8)	217 (85.8)	0.8453
INOCA Adversely affected your sex life	15 (57.7)	149 (58.9)	0.9518
Work and disability after onset of symptoms			
INOCA Adversely affecting work life	21 (80.8)	184 (72.7)	0.7345
Reduced Work hours due to INOCA symptoms	18 (69.2)	148 (58.5)	0.6028
Retired early because of INOCA	10 (38.5)	130 (51.4)	0.4540
Changed job/roles for less stressful position due to INOCA symptoms	7 (26.9)	104 (41.1)	0.3354
Changed job/roles resulting in lower pay due to INOCA symptoms	6 (23.1)	90 (35.6)	0.3539
Applied for disability because of INOCA symptoms	11 (42.3)	103 (40.7)	0.9191
Successful Application for disability benefits	9 (34.6)	79 (31.2)	0.8006

Legend: DASI = Duke Activity Status Index, METs = metabolic equivalents, INOCA = ischemia with no obstructive coronary artery disease.

## Data Availability

The data presented in this study are available from the corresponding author upon reasonable request.

## Supplementary Materials

The following supporting information can be downloaded at: https://www.mdpi.com/article/10.3390/jcm12175646/s1, Table S1. Baseline characteristics. Table S2. Symptoms, Triggers. Table S3. Referral Patterns & Evaluation.
