# Supplementary material for: Sex Differences in Quality of Life in Patients with Ischemia with No Obstructive Coronary Artery Disease (INOCA): A Patient Self-Report Retrospective Survey from INOCA International
Source: J Clin Med. 2023 Aug 30;12(17):5646. doi: 10.3390/jcm12175646 (PMC10488627; doi:10.3390/jcm12175646)
Supplement: Supplementary file 1 [file jcm-12-05646-s001.zip › jcm-2512781-supplementary.pdf]

Supplementary Material

Table S1. Baseline characteristics

	Men	Women
**Total (n)**	26	253
**Established Diagnoses (n, %)**		
Coronary Microvascular Disease	15 (57.7)	167 (66.0)
Coronary Artery Spasm	13 (50.0)	134 (53.0)
Nonobstructive atherosclerosis	3 (11.5)	14 (5.5)
Heart Failure with Preserved Ejection Fraction	0	13 (5.1)
Takotsubo Cardiomyopathy/Stress Cardiomyopathy	0	13 (5.1)
Not given any diagnosis aside from INOCA	3 (11.5)	21 (8.3)
Unknown	1 (3.8)	12 (4.7)
MINOCA	4 (15.4)	62 (24.5)
**Age at INOCA diagnosis (years)(n, %)**		
< 30	2 (7.7)	6 (2.4)
30 -40	4 (15.4)	25 (9.9)
40-50	7 (26.9)	68 (26.9)
50 -60	8 (30.8)	106 (41.9)
> 60	5 (19.2)	48 (19.0)
**Comorbidities (n, %)**		
Migraines/ frequent headaches	11 (42.3)	127 (50.2)
Raynaud's	4 (15.4)	60 (23.7)
Thyroid disorder	0	64 (25.3)
Rheumatoid Arthritis	2 (7.7)	14 (5.5)
Lupus/ systemic lupus erythematosus	0	4 (1.6)
Other autoimmune disorder	1 (3.8)	63 (24.9)
History of stroke	2 (7.7)	8 (3.2)
Kidney disease	1 (3.8)	14 (5.5)
None	14 (53.8)	62 (24.5)

Table S1 shows baseline characteristic stratified by sex. There were no statistical differences in baseline characteristic between men and women.

Legend: INOCA -Ischemia with No Obstructive Coronary Artery Disease

MINOCA- Myocardial Infarction with No Obstructive Coronary Artery Disease

Table S2: Symptoms, Triggers

	Men	Women
N	26	253
**Years with INOCA Symptoms (n, %)**		
< 1 Year	3 (11.5)	31 (12.3)
1-5 Years	18 (69.2)	99 (39.1)
5-10 Years	2 (7.7)	60 (23.7)
10-20 Years	2 (7.7)	43 (17.0)
> 20 Years	1 (3.8)	20 (7.9)
**Time From Symptom Onset to INOCA Dx**		
<1 Month	0	24 (9.5)
1 Month-1Year	9 (34.6)	80 (31.6)
1-3 Years	8 (30.8)	57 (22.5)
3-10 Years	6 (23.1)	66 (26.1)
>10 Years	3 (11.5)	26 (10.3)
**Clinical Assessment of Symptoms**		
Told that symptoms were not Cardiac	20 (76.9)	198 (78.3)
Seen in ED for symptoms + Discharged without Treatment	20 (76.9)	175 (69.2)
Told that symptoms although symptoms of INOCA are unpleasant, you cannot die from it or have a heart attack	13 (50.0)	172 (68.0)
Had ever called an ambulance for symptoms	13 (50.0)	152 (60.1)
**Ambulance response to INOCA Symptoms**		
Taken to hospital + ECG + Monitor	13 (50.0)	134 (52.3)
No ambulance dispatched	3 (11.5)	6 (2.4)
Assessed by ambulance crew but not taken to hospital	2 (7.7)	36 (14.2)
Taken to hospital but no ECG or cardiac monitor performed	1 (3.8)	22 (8.7)
Ambulance crew understood the diagnosis of INOCA	2 (7.7)	22 (8.7)
Ambulance crew did not understand the diagnosis of INOCA	3 (11.5)	71 (28.1)
I never had to call an ambulance	11 (42.3)	85 (33.6)
I do not call the ambulance because they do not take my symptoms seriously	3 (11.5)	39(15.4)

Table S2 continued

	Men	Women
**Symptoms**	**26**	**253**
General: Fatigue/Exhaustion, Sweats	24 (92.3)	228 (90.1)
Cardiovascular		
Chest pain/chest pressure/chest discomfort	26 (100)	249 (98.4)
Palpitations	14 (53.8)	166 (65.6)
Shortness of Breath	19 (73.1)	219 (86.7)
Back, shoulder, arm, neck, jaw pain	20 (76.9)	180 (71.1)
Neurologic: Confusion, brain fog, vision changes, light headedness, dizziness	18 (69.2)	187 (73.9)
Gastrointestinal: Nausea, reflux- like symptoms	8 (30.8)	137 (54.2)
Other	3 (11.5)	42 (16.6)
Triggers:		
Stress	22 (84.6)	215 (85.0)
Exercise/ Exertion	17 (65.4)	200 (79.1)
Excitement or High Emotional State/Anger	17 (65.4)	188 (74.3)
Cold Weather	13 (50)	164 (64.8)
Change in temperature or weather change	10 (38.5)	134 (53.0)
Triggered during Menstruation	-	48 (19.0)
Other	7 (26.9)	63 (24.9)
No known triggers	3 (11.5)	15 (5.9)

Table S2 shows symptoms and triggers stratified by sex. There were no statistical differences in symptoms and triggers between men and women.

Legend: ECG electrocardiogram; INOCA Ischemia with No Obstructive Coronary Artery Disease

Table S3: Referral Patterns & Evaluation

	Men	Women
**Prior to the Diagnosis of INOCA**		
Told symptoms were due to GERD	14 (53.8)	143 (56.5)
Underwent Endoscopy of Assess for GERD	11 (42.3)	82 (32.4)
Told symptoms were not Cardiac	20 (76.9)	197 (77.9)
Referred to a Psychiatrist for Symptoms	10 (38.5)	83 (32.8)
Rx for Antidepressant/Antianxiety	12 (46.2)	110 (43.5)
Seen in the ED for symptoms of INOCA and discharged	20 (76.9)	175 (69.2)
**Total Consults Seen Prior to INOCA Diagnosis**		
Diagnosed Right away	0	18 (7.4)
1-2 Additional consults	9 (34.6)	75 (29.8)
3-5 Additional consults	12 (46.2)	93 (36.0)
>5 Additional consults	5 (19.2)	67 (26.7)
**Non-invasive imaging**		
ECG	24 (92.3)	237 (93.7)
Echocardiogram	22 (84.6)	229 (90.5)
Exercise Stress Test	18 (69.2)	182 (71.9)
Stress Echocardiogram	11 (42.3)	133 (52.7)
CT Angiogram	15 (57.7)	119 (47.0)
Cardiac MRI	13 (50.0)	124 (49.0)
PET Scan	2 (7.7)	29 (11.5)
**Invasive Imaging**		
Cardiac Catheterization	16 (61.5)	168 (66.4)
Cardiac Catheterization with Acetylcholine testing	8 (30.8)	88 (34.8)
**# of Cardiologists Consulted for INOCA**		
1	5 (19.2)	53 (20.9)
2	6 (23.1)	68 (26.9)
≥3	15 (57.7)	132 (52.2)
**Finding a Specialist Aware of INOCA**		
Self-Referred	6 (23.1)	97 (38.3)
Referred by Cardiologist	6 (23.1)	38 (15.0)
Referred by Family Doctor or other doctor	1 (3.8)	17(6.7)
Never under the care of a specialist aware of INOCA	13 (50)	101 (39.9)
Currently under the care of a specialist aware of INOCA	12 (46.2)	131 (51.8)

Table S3 shows referral patterns and evaluation of symptoms stratified by sex. There were no statistical differences in referral patterns or evaluation between men and women.

Legend: CT computed tomography; ECG electrocardiogram; GERD Gastroesophageal Reflux Disease, NOCA Ischemia with No Obstructive Coronary Artery Disease, MRI magnetic resonance imaging; PET positron emission tomography.

Survey:

INOCA SURVEY

We are interested in how living with INOCA (Ischemia with No Obstructive Coronary Arteries) has impacted your medical care, health and life. Your responses will remain anonymous. Thank you for your time in responding to our questions.

Question 1

Do you have Ischemia with No Obstructive Coronary Arteries (INOCA)?

☐ Yes

☐ No

(if No, no further questions)

Question 2

Would you say that your general health is:

☐ Excellent

☐ Very Good

☐ Good

☐ Fair

☐ Poor

Question 3

Which of the following forms of INOCA were you diagnosed with? (Check all that apply)

☐ Coronary Microvascular Dysfunction

☐ Coronary Artery Spasm

☐ Nonobstructive Atherosclerosis

☐ Heart Failure with Preserved Ejection Fraction (HFpEF)

☐ Takutsubo's Syndrome (also known as Stress Cardiomyopathy/"Broken Heart" Syndrome)

☐ I was not given a diagnosis aside from INOCA

☐ I don't know

Question 4

How long did it take from the onset of your symptoms to getting a diagnosis of INOCA?

☐ Less than 1 months

☐ 1-12 months

☐ 1-3 years

☐ 3-5 years

☐ 5-10 years

☐ >10 years

Question 5

Prior to your diagnosis of INOCA were you ever told your symptoms were due to Reflux or GERD (gastroesophageal reflux disease)?

☐ Yes

☐ No

Question 6

Prior to your diagnosis of INOCA did you undergo an endoscopy to assess for reflux/GERD based on your symptoms?

☐ Yes

☐ No

Question 7

Prior to your diagnosis of INOCA were you ever told your symptoms were not cardiac?

☐ Yes

☐ No

Question 8

Prior to your diagnosis of INOCA were you seen in the Emergency Room/A&E for your symptoms of INOCA and discharged without any treatment?

☐ Yes

☐ No

Question 9

Prior to your onset of symptoms of INOCA, which of the following could you **previously** do? (Check All That Apply)

☐ Take Care of Yourself (ie. dress, eat, bathe, use toilet)

☐ Walking Indoors

☐ Climb a flight of stairs or walk up a hill

☐ Run a Short Distance

☐ Do light work around the house (ie. dusting, washing dishes)

☐ Do moderate work around the house (ie. vacuuming, sweeping floors, carrying groceries)

☐ Do heavy work around the house (ie. scrubbing floors, lifting or moving heavy furniture)

☐ Do yardwork (ie. raking leaves, weeding, pushing a lawn mower)

☐ Have Sexual Relations

☐ Participate in Moderate Recreational Activities (ie. golf, bowling, doubles tennis, throwing baseball, kicking football)

☐ Participate in Strenous Sports (ie. swimming, singles tennis, football, basketball, skiing)

Question 10

With your diagnosis of INOCA, which of the following can you **currently** do? (Check All That Apply)

☐ Take Care of Yourself (ie. dress, eat, bathe, use toilet)

☐ Walking Indoors

☐ Climb a flight of stairs or walk up a hill

☐ Run a Short Distance

☐ Do light work around the house (ie. dusting, washing dishes)

☐ Do moderate work around the house (ie. vacuuming, sweeping floors, carrying groceries)

☐ Do heavy work around the house (ie. scrubbing floors, lifting or moving heavy furniture)

☐ Do yardwork (ie. raking leaves, weeding, pushing a lawn mower)

☐ Have Sexual Relations

☐ Participate in Moderate Recreational Activities (ie. golf, bowling, doubles tennis, throwing baseball, kicking football)

☐ Participate in Strenous Sports (ie. swimming, singles tennis, football, basketball, skiing)

Question 11

How many consultants/specialists/doctors did you see prior to your diagnosis of INOCA?

☐ 0 (meaning diagnosed right away)

☐ 1-2

☐ 3-5

☐ >5

Question 12

How many cardiologists have you consulted for treatment of your INOCA?

☐ 1

☐ 2

☐ 3-5

☐ >5

Question 13

Prior to your diagnosis of INOCA were you ever referred to a psychiatrist for your symptoms or was such a referral suggested to you by your doctor?

☐ Yes

☐ No

Question 14

Have you ever been started on, or been recommended to start, an antidepressant or antianxiety medication for your INOCA symptoms?

☐ Yes

☐ No

Question 15

Are you under the care of a specialist in INOCA?

☐ Yes

☐ No

☐ Awaiting Initial Appointment

☐ I Don't Know

Question 16

If you under the care of a specialist in INOCA, how did you get to them?

☐ Self-Referred (I found the specialist myself)

☐ My Family Doctor/GP referred me to the INOCA specialist

☐ Another cardiologists referred me to the INOCA Specialist

☐ Another doctor referred me to the INOCA Specialist

☐ AI have never been under the care of an INOCA Specialist

Question 17

How many years have you had symptoms of INOCA for?

☐ Less than 1 year

☐ 1-5 years

☐ 5-10 years

☐ 10-20 years

☐ >20 years

Question 17

At What Age were you Diagnosed with INOCA?

☐ Less than 30

☐ 30-40

☐ 40-50

☐ 50-60

☐ 60-70

☐ >70 years

Question 18

Have you ever had a Heart Attack?

☐ Yes

☐ No

☐ Unsure

Question 19

Have you ever been told that although your symptoms of INOCA may be unpleasant, you cannot die from it and cannot have a heart attack?

☐ Yes

☐ No

Question 20

Have you ever had to call an Ambulance for your symptoms of INOCA?

☐ Yes

☐ No

Question 21

When you have called an Ambulance for your symptoms of INOCA, have you experienced any of the following? (choose all that apply)

☐ Taken to the Hospital and Cardiac Monitor Attached and ECG performed

☐ No Ambulance dispatched

☐ Assessed by Ambulance Crew but not taken to the hospital

☐ Taken to the Hospital but No Cardiac Monitor or ECG performed despite symptoms

☐ Ambulance Crew Understood the Diagnosis of INOCA

☐ Ambulance Crew DID NOT Understand the Diagnosis of INOCA

☐ I have never had to call an Ambulance

☐ I do not call the Ambulance because they do not take my symptoms seriously

---

Question 22

As a patient living with INOCA, do you know when to call for an ambulance or go to the hospital for your INOCA symptoms?

☐ Yes

☐ No

Question 23

Which diagnostic tests have you had related to your INOCA symptoms? (Check all that apply)

☐ ECG
☐ Echocardiogram (also called Echo)
☐ Exercise Stress Test

☐ Stress Echocardiogram (Also called Stress Echo)

☐ CT Angiogram

☐ Cardiac MRI

☐ PET Scan

☐ Cardiac Catheterization (Also called Angiogram)

☐ Cardiac Catheterization (Also called Angiogram) with Acetylcholine Testing

☐ None of the Above

Question 24

Which symptoms do you experience related to INOCA? (Check all that apply)

☐ Chest Pain/Chest Pressure/Chest Discomfort
☐ Fatigue/Exhaustion
☐ Shortness of Breath

☐ Back Pain

☐ Shoulder or Arm Pain or Pressure

☐ Neck/Jaw Pain

☐ Palpitations/Racing of the heart

☐ Sweats

☐ Lightheadedness, Dizzyness

☐ Nausea, reflux-like symptoms

☐ Confusion, Brain Fog

☐ Vision Changes

☐ Other

Question 25

Have You Ever Left any Doctor’s Appointment and come away thinking they did not understand INOCA?

☐ All the Time

☐ Often

☐ Occasionally

☐ Never

Question 26

Have You Ever Had to Stop Working because of INOCA?

☐ Yes
☐ No

Question 27

Did You Had to Retire Early because of INOCA?

☐ Yes
☐ No

Question 28

Have You Ever Had to Reduce Working Hours because of INOCA?

☐ Yes
☐ No

Question 29

Have You Ever Had to Change Jobs or Roles for a Less Stressful Position because of your symptoms from INOCA?

☐ Yes
☐ No

Question 30

Have You Ever Had to Change Jobs or Roles that Resulted in Lower Pay Because of your Symptoms with INOCA?

☐ Yes
☐ No

Question 31

Have You Ever Had to Apply for Disability Benefits because of your symptoms with INOCA?

☐ Yes
☐ No

Question 32

If You Had to Apply for Disability Benefits because of your symptoms with INOCA, was your application successful?

☐ Yes
☐ No
☐ I have never applied for disability benefits

Question 33

Do You Ever Worry about being home alone?

☐ Yes
☐ No

Question 34

Do You Ever Worry about going out alone?

☐ Yes
☐ No

Question 35

Do You Drive?

☐ Yes,
☐ No, stopped due to INOCA symptoms
☐ Never Drove

Question 36

Did you have any of the following conditions during pregnancy? (check all that apply)

☐ Hypertension During pregnancy

☐ Preeclampsia or Eclampsia

☐ Gestational Diabetes

☐ Preterm Delivery

☐ Miscarriage

☐ Does Not Apply To Me, I have Never Been Pregnant

Question 37

Do you have any of the following conditions? (check all that apply)

☐ Migraines/ Frequent Headaches

☐ Raynaud's

☐ Thyroid Disorder

☐ Rheumatoid Arthritis

☐ Lupus/ Systemic Lupus Erythematosus

☐ Other Autoimmune Disorder

☐ History of Stroke

☐ Kidney Disease

☐ None

Question 38

Do You Have Any of the Following Triggers for Your Symptoms of INOCA?

☐ Stress

☐ Exercise/Exertion

☐ Excitement or High Emotional State/Anger

☐ Cold Weather

☐ Change in Temperature or Weather Change

☐ Triggered during Menstruation

☐ Other

☐ No Known Triggers

Question 39

Did Your Symptoms Change at Menopause?

☐ Yes

☐ No

☐ Unsure

☐ Have not Undergone Menopause Yet

☐ Male (Not Applicable)

Question 40

Has INOCA Adversely Affected Your Home Life?

☐ Yes

☐ No

Question 41

Has INOCA Adversely Affected Your Relationship with Your Partner/Spouse?

☐ Yes

☐ No

☐ Not applicable

Question 42

Has INOCA Adversely Affected Your Work Life?

☐ Yes

☐ No

Question 43

Has INOCA Adversely Affected Your Social Life?

☐ Yes

☐ No

Question 44

Has INOCA Adversely Affected Your Sex Life?

☐ Yes

☐ No

☐ Not applicable

Question 45

Has INOCA Adversely Affected Your Mental Health?

☐ Yes

☐ No

Question 46

Has INOCA Negatively Affected Your Outlook on Life?

☐ Yes

☐ No

Question 47

Thinking about your physical health, which includes physical illness and injury, for how many days during the past 30 days was your physical health not good?

☐ __ (no number >30 will be accepted)

Question 48

Thinking about your mental health, which includes stress, depression and problems with emotions, for how many days during the past 30 days was your mental health not good?

☐ __(no number >30 will be accepted)

Question 49

During the past 30 days, for how many days did poor physical health or mental health, keep you from doing your usual activities, such as self-care, work or recreation?

☐ __(no number >30 will be accepted)

IF YOU WOULD LIKE TO SHARE ANY OTHER COMMENTS WITH YOU ABOUT YOUR EXPERIENCE LIVING WITH INOCA, PLEASE FEEL FREE TO WRITE ANY COMMENTS HERE:
